# Non-invasive detection of mutagenic exposure to aristolochic acid using ultra-deep sequencing of blood and urine samples

**DOI:** 10.64898/2026.10.02.26363145

**Published:** 2026-10-07

**Authors:** Peter T. Nguyen, Arnoud Boot, Po-Hung Lin, Sanskruti Sharma, Willie Yu, Mo Liu, Nanhai Jiang, Elizabeth Schmidt, Charles C. Valentine, Valerie Chew, Jesse J. Salk, Fang Yin Lo, Jacob See Tong Pang, Steven G. Rozen, Maria Zhivagui

## Abstract

Aristolochic acids (AAs) are potent nephrotoxins and carcinogens found in certain herbal remedies, driving a high burden of urothelial and liver malignancies globally. Currently, at a genomic level, AA exposure is detected retrospectively via DNA sequencing of clonal tumors, which limits opportunities to detect the mutational imprints of the exposure before tumor formation, when risk stratification may be possible. In a cohort of 12 urothelial carcinoma of the urinary tract patients, we profiled matched tumor and normal tissues alongside peripheral blood, urine sediments, and sorted leukocyte fractions. Rather than seeking tumor-derived clonal mutations, we instead interrogate non-clonal mutations associated with AA exposure. Here, we demonstrate that AA-induced mutagenesis can be non-invasively identified using ultra-deep duplex sequencing (DS), with the canonical AA signature, SBS22a, recovered in the blood and urine from patients harboring AA-positive tumors, but absent in AA-negative tumors. Single-cell WGS and DS of fractionated immune cells localized the SBS22a signal to the adaptive lymphoid compartment, identifying these long-lived cells as a systemic reservoir of mutational history. These findings show that a urothelial carcinogen leaves a durable, cell-type-resolved record in long-lived circulating lymphocytes, and establish a proof-of-concept framework for non-invasive carcinogen exposure surveillance.

## Full Text Availability

The license terms selected by the author(s) for this preprint version do not permit archiving in PMC. The full text is available from the preprint server.

